# Supplementary material for: Meiotic nuclear pore complex remodeling provides key insights into nuclear basket organization
Source: J Cell Biol. 2022 Dec 14;222(2):e202204039. doi: 10.1083/jcb.202204039 (PMC9754704; doi:10.1083/jcb.202204039)
Supplement: Table S1 — lists the strains used in this study. [file JCB_202204039_TableS1.docx]

**Table S1. Strains used in this study.**

| **Strain** | **Genotype** |
| --- | --- |
| SK1  Background | *ho::LYS2 lys2 ura3 leu2::hisG his3::hisG trp1::hisG* |
| UB15  Wild type | *MAT***a** */MATalpha* |
| UB11513 | *MAT***a** */MATalpha* *HTB1-mCherry::HISMX6/HTB1-mCherry::HISMX6 Nup170-GFP::KanMX/Nup170-GFP::KanMX* |
| UB13499 | *MAT***a** */MATalpha Nup120-GFP::KanMX/Nup120-GFP::KanMX HTB1-mCherry::HISMX6/HTB1-mCherry::HISMX6* |
| UB13503 | *MAT***a** */MATalpha Pom34-GFP::KanMX/Pom34-GFP::KanMX* *HTB1-mCherry::HISMX6/HTB1-mCherry::HISMX6* |
| UB13509 | *MAT***a** */MATalpha Nup49-GFP::KanMX/Nup49::KanMX HTB1-mCherry::HISMX6/HTB1-mCherry::HISMX6* |
| UB14646 | *MAT***a** */MATalpha Nup60-GFP::KanMX/ Nup60-GFP::KanMX HTB1-mCherry::HISMX6/HTB1-mCherry::HISMX6* |
| UB14648 | *MAT***a** */MATalpha Mlp1-GFP-KanMX/Mlp1-GFP-KanMX HTB1-mCherry-HISMX6/HTB1-mCherry-HISMX6* |
| UB15303 | *MAT***a** */MATalpha Nup1-GFP::KanMX/ Nup1-GFP::KanMX HTB1-mCherry::HISMX6/HTB1-mCherry::HISMX6* |
| UB15305 | *MAT***a** */MATalpha Nup2-GFP::KanMX/ Nup2-GFP::KanMX HTB1-mCherry::HISMX6/HTB1-mCherry::HISMX6* |
| UB20080 | *MAT***a** */MATalpha Nup2-GFP-KanMX/Nup2-GFP-KanMX Pom34-mCherry-HISMX/Pom34-mCherry-HISMX* |
| UB21079 | *MAT***a** */MATalpha Nup84-GFP::KanMX/Nup84-GFP::KanMX Pom34-mCherry-HISMX/Pom34-mCherry-HISMX* |
| UB24613 | *MAT***a** */MATalpha Seh1-GFP-KanMX/Seh1-GFP-KanMX HTB1-mCherry-HISMX6/HTB1-mCherry-HISMX6* |
| UB25727 | *MAT***a** */MATalpha Nup1-delta2-32-GFP-KanMX/Nup1-delta2-32-GFP-KanMX HTB1-mCherry-HISMX6/HTB1-mCherry-HISMX6* |
| UB25731 | *MAT***a** */MATalpha Nup60-delta2-47-GFP-KanMX/Nup60-delta2-47-GFP-KanMX HTB1-mCherry-HISMX6/HTB1-mCherry-HISMX6* |
| UB25843 | *MAT***a** */MATalpha Fpr1::HygMX/Fpr1::HygMX FKBP12-Nup60 (unmarked)/FKBP12-Nup60 (unmarked) Seh1-FRB-KanMX/Seh1-FRB-KanMX Nup2-GFP-KanMX/Nup2-GFP-KanMX Pom34-mCherry-HISMX/Pom34-mCherry-HISMX* |
| UB27143 | *MAT***a** */MATalpha Fpr1::HygMX/Fpr1::HygMX FKBP12-Nup60 (unmarked)/FKBP12-Nup60 (unmarked) Seh1-FRB-KanMX/Seh1-FRB-KanMX Nup1-GFP-KanMX/Nup1-GFP-KanMX Pom34-mCherry-HISMX/Pom34-mCherry-HISMX* |
| UB27189 | *MAT***a** */MATalpha Nup60-I36R-GFP-KanMX/Nup60-I36R-GFP-KanMX HTB1-mCherry-HISMX6/HTB1-mCherry-HISMX6* |
| UB27298 | *MAT***a** */MATalpha FKBP12-Nup60-GFP-KanMX/FKBP12-Nup60-GFP-KanMX Pom34-mCherry-HISMX/Pom34-mCherry-HISMX Fpr1::HygMX/Fpr1::HygMX Seh1-FRB-KanMX/Seh1-FRB-KanMX*  [Diploid 1] |
| UB27299 | *MAT***a** */MATalpha FKBP12-Nup60-GFP-KanMX/FKBP12-Nup60-GFP-KanMX Pom34-mCherry-HISMX/Pom34-mCherry-HISMX Fpr1::HygMX/Fpr1::HygMX Seh1-FRB-KanMX/Seh1-FRB-KanMX*  [Diploid 2] |
| UB27725 | *MAT***a** */MATalpha Fpr1::HygMX/Fpr1::HygMX FKBP12-Nup60 (unmarked)/FKBP12-Nup60 (unmarked) Seh1-FRB-KanMX/Seh1-FRB-KanMX Mlp1-GFP-KanMX/Mlp1-GFP-KanMX Pom34-mCherry-HISMX/Pom34-mCherry-HISMX* |
| UB28201 | *MAT***a** */MATalpha SPC42-mCherry::NAT/SPC42-mCherry::NAT Nup60-GFP-KanMX/Nup60-GFP-KanMX* |
| UB28211 | *MAT***a** */MATalpha cdc20::pCLB2-CDC20::TRP1/cdc20::pCLB2-CDC20::TRP1 Nup60-GFP-KanMX/Nup60-GFP-KanMX HTB1-mCherry-HISMX6/HTB1-mCherry-HISMX6* |
| UB28213 | *MAT***a** */MATalpha cdc20::pCLB2-CDC20::TRP1/cdc20::pCLB2-CDC20::TRP1 Nup60-delta2-47-GFP-KanMX/Nup60-delta2-47-GFP-KanMX HTB1-mCherry-HISMX6/HTB1-mCherry-HISMX6* |
| UB28492 | *MAT***a** */MATalpha cdc5::pCLB2-CDC5::KanMX6/cdc5::pCLB2-CDC5::KanMX6 Nup60-GFP-KanMX/Nup60-GFP-KanMX HTB1-mCherry-HISMX6/HTB1-mCherry-HISMX6* |
| UB28494 | *MAT***a** */MATalpha cdc5::pCLB2-CDC5::KanMX6/cdc5::pCLB2-CDC5::KanMX6 Nup60-delta2-47-GFP-KanMX/Nup60-delta2-47-GFP-KanMX HTB1-mCherry-HISMX6/HTB1-mCherry-HISMX6* |
| UB28614 | *MAT***a** */MATalpha cdc20::pCLB2-CDC20::TRP1/cdc20::pCLB2-CDC20::TRP1 cdc5::pCLB2-CDC5::KanMX6/cdc5::pCLB2-CDC5::KanMX6 Nup60-GFP-KanMX/Nup60-GFP-KanMX HTB1-mCherry-HISMX6/HTB1-mCherry-HISMX6* |
| UB28616 | *MAT***a** */MATalpha cdc20::pCLB2-CDC20::TRP1/cdc20::pCLB2-CDC20::TRP1 cdc5::pCLB2-CDC5::KanMX6/cdc5::pCLB2-CDC5::KanMX6 Nup60-delta2-47-GFP-KanMX/Nup60-delta2-47-GFP-KanMX HTB1-mCherry-HISMX6/HTB1-mCherry-HISMX6* |
| UB29069 | *MAT***a** */MATalpha ndt80::LEU2/ndt80::LEU2 trp1::pCUP1-Cdc5[KD]-3xFLAG-10xHIS::TRP1/trp1::pCUP1-Cdc5[KD]-3xFLAG-10xHIS::TRP1 Nup60-GFP-KanMX/Nup60-GFP-KanMX HTB1-mCherry-HISMX6/HTB1-mCherry-HISMX6* |
| UB29071 | *MAT***a** */MATalpha ndt80::LEU2/ndt80::LEU2 trp1::pCUP1-Cdc5-3xFLAG-10xHIS::TRP1/trp1::pCUP1-Cdc5-3xFLAG-10xHIS::TRP1 Nup60-delta2-47-GFP-KanMX/Nup60-delta2-47-GFP-KanMX HTB1-mCherry-HISMX6/HTB1-mCherry-HISMX6* |
| UB29073 | *MAT***a** */MATalpha ndt80::LEU2/ndt80::LEU2 trp1::pCUP1-Cdc5[KD]-3xFLAG-10xHIS::TRP1/trp1::pCUP1-Cdc5[KD]-3xFLAG-10xHIS::TRP1 Nup60-delta2-47-GFP-KanMX/Nup60-delta2-47-GFP-KanMX HTB1-mCherry-HISMX6/HTB1-mCherry-HISMX6* |
| UB29129 | *MAT***a** */MATalpha ndt80::LEU2/ndt80::LEU2 trp1::pCUP1-Cdc5-3xFLAG-10xHIS::TRP1/trp1::pCUP1-Cdc5-3xFLAG-10xHIS::TRP1 Nup60-GFP-KanMX/Nup60-GFP-KanMX HTB1-mCherry-HISMX6/HTB1-mCherry-HISMX6* |
| UB29249 | *MAT***a** */MATalpha cdc20::pCLB2-CDC20::TRP1/cdc20::pCLB2-CDC20::TRP1 cdc5::pCLB2-CDC5::KanMX6/cdc5::pCLB2-CDC5::KanMX6 SPC42-mCherry::NAT/SPC42-mCherry::NAT Nup60-GFP-KanMX/Nup60-GFP-KanMX* |
| UB29251 | *MAT***a** */MATalpha cdc5::pCLB2-CDC5::KanMX6/cdc5::pCLB2-CDC5::KanMX6 SPC42-mCherry::NAT/SPC42-mCherry::NAT Nup60-GFP-KanMX/Nup60-GFP-KanMX* |
| UB29255 | *MAT***a** */MATalpha cdc20::pCLB2-CDC20::TRP1/cdc20::pCLB2-CDC20::TRP1 cdc5::pCLB2-CDC5::KanMX6/cdc5::pCLB2-CDC5::KanMX6 SPC42-mCherry::NAT/SPC42-mCherry::NAT Nup60-delta2-47-GFP-KanMX/Nup60-delta2-47-GFP-KanMX* |
| UB29257 | *MAT***a** */MATalpha cdc5::pCLB2-CDC5::KanMX6/cdc5::pCLB2-CDC5::KanMX6 SPC42-mCherry::NAT/SPC42-mCherry::NAT Nup60-delta2-47-GFP-KanMX/Nup60-delta2-47-GFP-KanMX* |
| UB29259 | *MAT***a** */MATalpha cdc20::pCLB2-CDC20::TRP1/cdc20::pCLB2-CDC20::TRP1 SPC42-mCherry::NAT/SPC42-mCherry::NAT Nup60-delta2-47-GFP-KanMX/Nup60-delta2-47-GFP-KanMX* |
| UB29253 | *MAT***a** */MATalpha cdc20::pCLB2-CDC20::TRP1/cdc20::pCLB2-CDC20::TRP1 SPC42-mCherry::NAT/SPC42-mCherry::NAT Nup60-GFP-KanMX/Nup60-GFP-KanMX* |
| UB29265 | *MAT***a** */MATalpha Nup60-S89A-GFP-KanMX/Nup60-S89A-GFP-KanMX HTB1-mCherry-HISMX6/HTB1-mCherry-HISMX6* |
| UB29267 | *MAT***a** */MATalpha Nup60-Cterm4A-GFP-KanMX/Nup60-Cterm4A-GFP-KanMX HTB1-mCherry-HISMX6/HTB1-mCherry-HISMX6* |
| UB29337 | *MAT***a** */MATalpha Fpr1::HygMX/Fpr1::HygMX Seh1-FRB-KanMX/Seh1-FRB-KanMX FKBP12-Mlp1-GFP-KanMX/FKBP12-Mlp1-GFP-KanMX Pom34-mCherry-HISMX/Pom34-mCherry-HISMX* |
| UB29358 | *MAT***a** */MATalpha Nup60-9A-GFP-KanMX/Nup60-9A-GFP-KanMX HTB1-mCherry-HISMX6/HTB1-mCherry-HISMX6* |
| UB29441 | *MAT***a** */MATalpha Nup60-Nterm3A-GFP-KanMX/Nup60-Nterm3A-GFP-KanMX HTB1-mCherry-HISMX6/HTB1-mCherry-HISMX6* |
| UB29443 | *MAT***a** */MATalpha Nup60-Nterm5A-GFP-KanMX/Nup60-Nterm5A-GFP-KanMX HTB1-mCherry-HISMX6/HTB1-mCherry-HISMX6* |
| UB29560 | *MAT***a** */MATalpha ndt80::LEU2/ndt80::LEU2 trp1::pCUP1-Cdc5-3xFLAG-10xHIS::TRP1/trp1::pCUP1-Cdc5-3xFLAG-10xHIS::TRP1 Nup60-S89A-GFP-KanMX/Nup60-S89A-GFP-KanMX HTB1-mCherry-HISMX6/HTB1-mCherry-HISMX6* |
| UB29562 | *MAT***a** */MATalpha ndt80::LEU2/ndt80::LEU2 trp1::pCUP1-Cdc5-3xFLAG-10xHIS::TRP1/trp1::pCUP1-Cdc5-3xFLAG-10xHIS::TRP1 Nup60-Cterm4A-GFP-KanMX/Nup60-Cterm4A-GFP-KanMX HTB1-mCherry-HISMX6/HTB1-mCherry-HISMX6* |
| UB29564 | *MAT***a** */MATalpha ndt80::LEU2/ndt80::LEU2 trp1::pCUP1-Cdc5-3xFLAG-10xHIS::TRP1/trp1::pCUP1-Cdc5-3xFLAG-10xHIS::TRP1 Nup60-9A-GFP-KanMX/Nup60-9A-GFP-KanMX HTB1-mCherry-HISMX6/HTB1-mCherry-HISMX6* |
| UB29636 | *MAT***a** */MATalpha ndt80::LEU2/ndt80::LEU2 trp1::pCUP1-Cdc5-3xFLAG-10xHIS::TRP1/trp1::pCUP1-Cdc5-3xFLAG-10xHIS::TRP1 Nup60-Nterm3A-GFP-KanMX/Nup60-Nterm3A-GFP-KanMX HTB1-mCherry-HISMX6/HTB1-mCherry-HISMX6* |
| UB29638 | *MAT***a** */MATalpha ndt80::LEU2/ndt80::LEU2 trp1::pCUP1-Cdc5-3xFLAG-10xHIS::TRP1/trp1::pCUP1-Cdc5-3xFLAG-10xHIS::TRP1 Nup60-Nterm5A-GFP-KanMX/Nup60-Nterm5A-GFP-KanMX HTB1-mCherry-HISMX6/HTB1-mCherry-HISMX6* |
| UB30166 | *MAT***a** */MATalpha Fpr1::HygMX/Fpr1::HygMX Seh1-FRB-KanMX/Seh1-FRB-KanMX FKBP12-Mlp1 (unmarked)/FKBP12-Mlp 1(unmarked) Nup1-GFP-KanMX/Nup1-GFP-KanMX Pom34-mCherry-HISMX/Pom34-mCherry-HISMX* |
| UB30168 | *MAT***a** */MATalpha Fpr1::HygMX/Fpr1::HygMX Seh1-FRB-KanMX/Seh1-FRB-KanMX FKBP12-Mlp1 (unmarked)/FKBP12-Mlp1 (unmarked) Nup2-GFP-KanMX/Nup2-GFP-KanMX Pom34-mCherry-HISMX/Pom34-mCherry-HISMX* |
| UB30174 | *MAT***a** */MATalpha Fpr1::HygMX/Fpr1::HygMX Seh1-FRB-KanMX/Seh1-FRB-KanMX FKBP12-Mlp1 (unmarked)/FKBP12-Mlp1 (unmarked) Nup60-GFP-KanMX/Nup60-GFP-KanMX Pom34-mCherry-HISMX/Pom34-mCherry-HISMX* |
| UB30327 | *MAT***a** */MATalpha cdc20::pCLB2-CDC20::TRP1/cdc20::pCLB2-CDC20::TRP1 Nup60-S89A-GFP-KanMX/Nup60-S89A-GFP-KanMX SPC42-mCherry::NAT/SPC42-mCherry::NAT* |
| UB30329 | *MAT***a** */MATalpha cdc20::pCLB2-CDC20::TRP1/cdc20::pCLB2-CDC20::TRP1 Nup60-Nterm3A-GFP-KanMX/Nup60-Nterm3A-GFP-KanMX SPC42-mCherry::NAT/SPC42-mCherry::NAT* |
| UB30331 | *MAT***a** */MATalpha cdc20::pCLB2-CDC20::TRP1/cdc20::pCLB2-CDC20::TRP1 Nup60-Nterm5A-GFP-KanMX/Nup60-Nterm5A-GFP-KanMX SPC42-mCherry::NAT/SPC42-mCherry::NAT* |
| UB30333 | *MAT***a** */MATalpha cdc20::pCLB2-CDC20::TRP1/cdc20::pCLB2-CDC20::TRP1 Nup60-Cterm4A-GFP-KanMX/Nup60-Cterm4A-GFP-KanMX SPC42-mCherry::NAT/SPC42-mCherry::NAT* |
| UB30438 | *MAT***a** */MATalpha cdc20::pCLB2-CDC20::TRP1/cdc20::pCLB2-CDC20::TRP1 Nup60-9A-GFP-KanMX/Nup60-9A-GFP-KanMX Spc42-mCherry/Spc42-mCherry* |
| UB30628 | *MAT***a** */MATalpha Nup60-delta2-47 (unmarked)/Nup60-delta2-47 (unmarked) Nup1-GFP-KanMX/Nup1-GFP-KanMX HTB1-mCherry-HISMX6/HTB1-mCherry-HISMX6* |
| UB30630 | *MAT***a** */MATalpha Nup60-delta2-47 (unmarked)/Nup60-delta2-47 (unmarked) Nup2-GFP-KanMX/Nup2-GFP-KanMX HTB1-mCherry-HISMX6/HTB1-mCherry-HISMX6* |
| UB30632 | *MAT***a** */MATalpha Nup60-delta2-47 (unmarked)/Nup60-delta2-47 (unmarked) Mlp1-GFP-KanMX/Mlp1-GFP-KanMX HTB1-mCherry-HISMX6/HTB1-mCherry-HISMX6* |
| UB30640 | *MAT***a** */MATalpha Nup60-I36R (unmarked)/Nup60-I36R (unmarked) Mlp1-GFP-KanMX/Mlp1-GFP-KanMX HTB1-mCherry-HISMX6/HTB1-mCherry-HISMX6* |
| UB31262 | *MAT***a** */MATalpha Nup60::HygMX/Nup60::HygMX Mlp1-GFP-KanMX/Mlp1-GFP-KanMX HTB1-mCherry-HISMX6/HTB1-mCherry-HISMX6* |
| UB31600 | *MAT***a** */MATalpha Nup60::HygMX/Nup60::HygMX Nup2-GFP-KanMX/Nup2-GFP-KanMX HTB1-mCherry-HISMX6/HTB1-mCherry-HISMX6* |
| UB31853 | *MAT***a** */MATalpha Nup60-9A (unmarked)/ Nup60-9A (unmarked)*  [Diploid 1] |
| UB31854 | *MAT***a** */MATalpha Nup60-9A (unmarked)/ Nup60-9A (unmarked)*  [Diploid 2] |
| UB31855 | *MAT***a** */MATalpha Nup60-delta2-47 (unmarked)/ Nup60-delta2-47 (unmarked)*  [Diploid 1] |
| UB31856 | *MAT***a** */MATalpha Nup60-delta2-47 (unmarked)/ Nup60-delta2-47 (unmarked)*  [Diploid 2] |
| UB32238 | *MAT***a** */MATalpha his3::pCup1-OsTIR1-F74G (codon optimized)::HIS3/his3::pCup1-OsTIR1-F74G (codon optimized)::HIS3 Nup60-3V5-IAA17-KanMX (Vinny linker)/Nup60-3V5-IAA17-KanMX (Vinny linker) ndt80::LEU2/ndt80::LEU2 Nup2-GFP-KanMX/Nup2-GFP-KanMX HTB1-mCherry-HISMX6/HTB1-mCherry-HISMX6* |
| UB32240 | *MAT***a** */MATalpha Nup60-3V5-IAA17-KanMX (Vinny linker)/Nup60-3V5-IAA17-KanMX (Vinny linker) ndt80::LEU2/ndt80::LEU2 Nup2-GFP-KanMX/Nup2-GFP-KanMX HTB1-mCherry-HISMX6/HTB1-mCherry-HISMX6* |
| UB32246 | *MAT***a** */MATalpha his3::pCup1-OsTIR1-F74G (codon optimized)::HIS3*  *his3::pCup1-OsTIR1-F74G (codon optimized)::HIS3 Nup60-3V5-IAA17-KanMX (Vinny linker)/Nup60-3V5-IAA17-KanMX (Vinny linker) ndt80::LEU2/ndt80::LEU2 Mlp1-GFP-KanMX/Mlp1-GFP-KanMX HTB1-mCherry-HISMX6/HTB1-mCherry-HISMX6* |
| UB32248 | *MAT***a** */MATalpha Nup60-3V5-IAA17-KanMX (Vinny linker)/Nup60-3V5-IAA17-KanMX (Vinny linker) ndt80::LEU2/ndt80::LEU2 Mlp1-GFP-KanMX/Mlp1-GFP-KanMX HTB1-mCherry-HISMX6/HTB1-mCherry-HISMX6* |
| UB34086 | *MAT***a** */MATalpha Pom34-GFP::KanMX/Pom34-GFP::KanMX HTB1-mCherry-HISMX6/HTB1-mCherry-HISMX6 Nup60-delta2-47 (unmarked)/Nup60-delta2-47 (unmarked)* |
| UB34088 | *MAT***a** */MATalpha HTB1-mCherry-HISMX6/HTB1-mCherry-HISMX6 Nup170-GFP::KanMX/Nup170-GFP::KanMX Nup60-delta2-47 (unmarked)/Nup60-delta2-47 (unmarked)* |
| UB34204 | *MAT***a** */MATalpha FKBP12-Nup60-GFP-KanMX/FKBP12-Nup60-GFP-KanMX Pom34-mCherry-HISMX/Pom34-mCherry-HISMX Fpr1::HygMX/Fpr1::HygMX*  [Diploid 1] |
| UB34205 | *MAT***a** */MATalpha FKBP12-Nup60-GFP-KanMX/FKBP12-Nup60-GFP-KanMX Pom34-mCherry-HISMX/Pom34-mCherry-HISMX Fpr1::HygMX/Fpr1::HygMX*  [Diploid 2] |
| UB34452 | *MAT***a** */MATalpha Nup2-RITE (GFP->mCherry)::HygMX/ Nup2-RITE (GFP->mCherry)::HygMX SPC42-mCherry::NAT/SPC42-mCherry::NAT HIS3::pGPD/TDH3-Cre-EBD78-His3/HIS3::pGPD/TDH3-Cre-EBD78-His3* |
| UB34454 | *MAT***a** */MATalpha Nup2-RITE (GFP->mCherry)::HygMX/Nup2-RITE (GFP->mCherry)::HygMX SPC42-mCherry::NAT/SPC42-mCherry::NAT* |
| YML 1110 | *MAT****a*** *ndt80Δ::NatMX4 ura3::pGPD-GAL4-ER-URA3* |
| YML 3993 | *MAT***a** */MATalpha ndt80Δ::NatMX4/ndt80Δ::NatMX4 ura3::pGPD-GAL4-ER-URA3/ura3::pGPD-GAL4-ER-URA3 trp1::pGAL1-CDC5-WT-eGFP-TRP1/trp1::pGAL1-CDC5-WT-eGFP-TRP1* |
| YML 3994 | *MAT***a** */MATalpha ndt80Δ::NatMX4/ndt80Δ::NatMX4 ura3::pGPD-GAL4-ER-URA3/ura3::pGPD-GAL4-ER-URA3 trp1::pGAL1-CDC5-KD-eGFP-TRP1/trp1::pGAL1-CDC5-KD-eGFP-TRP1* |
| YML 6662 | *MAT***a** */MATalpha NUP60-myc9::KITRP1/NUP60-myc9::KITRP1* |
| YML 6665 | *MAT***a** */MATalpha NUP60-myc9::KITRP1/NUP60-myc9::KITRP1 cdc20::P_CLB2_-CDC20::KanMX6/cdc20::P_CLB2_-CDC20::KanMX6* |
| YML 6664 | *MAT***a** */MATalpha NUP60-myc9::KITRP1/NUP60-myc9::KITRP1 cdc20::P_CLB2_-CDC20::KanMX6/cdc20::P_CLB2_-CDC20::KanMX6 cdc5::P_CLB2_-CDC5::HphMX4/cdc5::P_CLB2_-CDC5::HphMX4* |
| YML 7956 | *MAT***a** */MATalpha NUP60^S89A^-myc9::KITRP1/NUP60^S89A^-myc9::KITRP1 cdc20::P_CLB2_-CDC20::KanMX6/cdc20::P_CLB2_-CDC20::KanMX6* |
| YML 7800 | *MAT***a** */MATalpha SLK19-myc9::KITRP1/SLK19-myc9::KITRP1 cdc20::P_CLB2_-CDC20::KanMX6/cdc20::P_CLB2_-CDC20::KanMX6* |
| YML 7801 | *MAT***a** */MATalpha SLK19-myc9::KITRP1/SLK19-myc9::KITRP1 cdc20::P_CLB2_-CDC20::KanMX6/cdc20::P_CLB2_-CDC20::KanMX6 cdc5::P_CLB2_-CDC5::HphMX4/cdc5::P_CLB2_-CDC5::HphMX4* |
| YML 8836 | *MAT***a** */MATalpha SWI6-myc9::KITRP1/SWI6-myc9::KITRP1 cdc20::P_CLB2_-CDC20::KanMX6/cdc20::P_CLB2_-CDC20::KanMX6* |
| YML 8837 | *MAT***a** */MATalpha SWI6-myc9::KITRP1/SWI6-myc9::KITRP1 cdc20::P_CLB2_-CDC20::KanMX6/cdc20::P_CLB2_-CDC20::KanMX6 cdc5::P_CLB2_-CDC5::HphMX4/cdc5::P_CLB2_-CDC5::HphMX4* |
| YML 12334 | *MAT****a****/MATalpha NUP60-myc9::KITRP1/NUP60-myc9::KITRP1 ndt80∆::HIS3/ndt80∆::HIS3 natNT2::pCUP1-1-CDC5/natNT2::pCUP1-1-CDC5* |
| fySLJ456 | *h-, Ppc89-mCherry-HygMX6, his3-D1, leu1-32, ura4-D18, ade6-M210* |
| fySLJ479 | *h+, Ppc89-mCherry-HygMX6, his3-D1, leu1-32, ura4-D18, ade6-M210* |
| fySLJ537 | *h-, Pom34-GFP-KanMX, Ppc89-mCherry-NatMX6, his3-DA, leu1-34, ura4-D18, ade6-M210* |
| fySLJ730 | *h-, Nup60-GFP-KanMX, his3-D1, leu1-32, ura4-D18, ade6-M210* |
| fySLJ745 | *h-, Nup60-GFP-KanMX, Ppc89-mCherry-NatMX6, his3-DA, leu1-34, ura4-D18, ade6-M210* |
| fySLJ840 | *h-, Nup61-GFP-KanMX, his3-D1, leu1-32, ura4-D18, ade6-M210* |
| fySLJ842 | *h-, Alm1-GFP-KanMX, his3-D1, leu1-32, ura4-D18, ade6-M210* |
| fySLJ867 | *h?, Alm1-GFP-KanMX, Ppc89-mCherry-NatMX6, his3-DA, leu1-34, ura4-D18, ade6-M210* |
| fySLJ870 | *h?, Nup61-GFP-KanMX, Ppc89-mCherry-NatMX6, his3-DA, leu1-34, ura4-D18, ade6-M210* |
| fySLJ989 | *h+, Nup124-GFP-KanMX, his3-DA, leu1-34, ura4-D18, ade6-M210* |
| fySLJ990 | *h+, Nup211-GFP-KanMX, his3-DA, leu1-34, ura4-D18, ade6-M210* |
| fySLJ1018 | *h-, Nup124-GFP-KanMX, Ppc89-mCherry-NatMX6, his3-DA, leu1-34, ura4-D18, ade6-M210* |
| fySLJ1019 | *h?, Nup211-GFP-KanMX, Ppc89-mCherry-NatMX6, his3-DA, leu1-34, ura4-D18, ade6-M210* |
| fySLJ1242 | *h-, Pom34-GFP-KanMX, Ppc89-mCherry-HygMX6, his3-DA, leu1-34, ura4-D18, ade6-M210* |
| fySLJ1243 | *h+, Pom34-GFP-KanMX, Ppc89-mCherry-HygMX6, his3-DA, leu1-34, ura4-D18, ade6-M210* |
