# Supplementary material for: Meiotic nuclear pore complex remodeling provides key insights into nuclear basket organization
Source: J Cell Biol. 2022 Dec 14;222(2):e202204039. doi: 10.1083/jcb.202204039 (PMC9754704; doi:10.1083/jcb.202204039)
Supplement: Table S2 — lists the plasmids used in this study. [file JCB_202204039_TableS2.docx]

**Table S2. Plasmids used in this study.**

| **Plasmid Name** | **Description** |
| --- | --- |
| pUB4/pSJ2445 | pFA6a-GFP(S65T)-KanMX6; *Addgene #39292* |
| pUB217 | pFA6a-HphNT1 |
| pUB595 | pFA6a-FRB-KanMX6 |
| pUB651 | pNH604-pGPD1-FKBP12-CTEV |
| pUB916 | pFA6a-mCherry-His3MX6 |
| pUB1305 | pL245-3V5-IAA17 |
| pUB1727 | Cas9-Nup1 gRNA4 Ura CEN plasmid |
| pUB1729 | Cas9-Nup60 gRNA4 Ura CEN plasmid |
| pUB1198 | RITE V5-LoxP-HA-GFP->LoxP-T7-mCherry |
| pUB2020 | Cas9-Mlp1 gRNA1 Ura CEN plasmid |
| pUB2047 | pCUP1-Cdc5-3xFLAG-10xHis Trp single integration vector |
| pUB2048 | pCUP1-Cdc5[KD]-3xFLAG-10xHis Trp single integration vector |
| pUB2120 | pCup-OsTIR-F74G His3 Integrating Vector |
| pML67 | pWZV87 (pUC19-Myc9-KITRP1) |
| pML118 | pAG304GAL-Cdc5WT-EGFP-TRP1 |
| pML120 | pAG304GAL-Cdc5KD-EGFP-TRP1 |
| pSJ2392 | pFA6a-mCherry-hphMX6; *Addgene #105156* |
