# Supplementary material for: Meiotic nuclear pore complex remodeling provides key insights into nuclear basket organization
Source: J Cell Biol. 2022 Dec 14;222(2):e202204039. doi: 10.1083/jcb.202204039 (PMC9754704; doi:10.1083/jcb.202204039)
Supplement: Table S3 — shows the primers used for deletion and C-terminal tagging in this study. [file JCB_202204039_TableS3.docx]

**Table S3. Primers used for deletion and C-terminal tagging in this study.**

| **Construct name** | **Forward primer** | **Reverse primer** |
| --- | --- | --- |
| Pom34-mCherry | TGCATATATGATGAACTCACAGTCCCCAAGGGGTAAAATA**CGGATCCCCGGGTTAATTAA** | TATATAGCTATGGAAAGTATTAAATGTTTTTTTGCTGTTT**GAATTCGAGCTCGTTTAAAC** |
| *fpr1Δ* | AACTCGAGTATAAGCAAAAAATCAATCAAAACAAGTAATA**cggatccccgggttaattaa** | TAAAAAGCAGAAAGGCGGCTCAATTGATAGTACTTTGCTT**GAATTCGAGCTCGTTTAAAC** |
| Seh1-FRB  Seh1-GFP | AAATGAATTTAAGTGTATGTCAGTAATTACTGCCCAACAA**CGGATCCCCGGGTTAATTAA** | AAGTACCAATATATAATGTTATGTATACATATATTCTTAT**GAATTCGAGCTCGTTTAAAC** |
| FKBP12-Nup60-GFP  Nup60^ΔAH^-GFP  Nup60^I36R^-GFP  Nup60^S89A^-GFP  Nup60^Nterm3A^-GFP  Nup60^Nterm5A^-GFP  Nup60^Cterm4A^-GFP  Nup60^9A^-GFP | TGAAAATAAAGTTGAGGCTTTCAAGTCCCTATATACCTTT**CGGATCCCCGGGTTAATTAA** | GGGCTATACGGTAATTATGTCACGGCTAAAATTTTCATTA**GAATTCGAGCTCGTTTAAAC** |
| Nup60-3V5-IAA17 | TGAAAATAAAGTTGAGGCTTTCAAGTCCCTATATACCTTT**gcggccgctctagaactagtgg** | GGGCTATACGGTAATTATGTCACGGCTAAAATTTTCATTA**ccccctcgaggtcgacggtatcg** |
| *nup60Δ* | ATCAAATAAGCACCGCAAGATATCCTAAAATCGACATCCA**CGGATCCCCGGGTTAATTAA** | GGGCTATACGGTAATTATGTCACGGCTAAAATTTTCATTA**GAATTCGAGCTCGTTTAAAC** |
| Mlp1-GFP  FKBP12-Mlp1-GFP | AGAAAAAGAAACCGATAAGGTGAATGACGAGAACAGTATA **cggatccccgggttaattaa** | AAGGTTTAGTTTGTATTGATCCCTTGTTTTTACTATCTCCT **GAATTCGAGCTCGTTTAAAC** |
| Nup1^ΔAH^-GFP | GGCGAACAGAAAGATTGCAAGAATGAGGCACTCTAAAAGG**CGGATCCCCGGGTTAATTAA** | TTCAGAAAAGCAACACAATACCTAATTACATAACCGATAT**GAATTCGAGCTCGTTTAAAC** |
| Nup2-RITE | ATTTACGAAAGCTATTGAAGATGCTAAAAAAGAAATGAAA**GGTGGATCTGGTGGATCT** | AGGGTTCTATTCTATTTAAAATTGTTAACTGTATTTACTC**TGATTACGCCAAGCTCG** |
| Nup60-myc9 | GCTTGGTTGATGAAAATAAAGTTGAGGCTTTCAAGTCCCTATATACCTTT**TCCGGTTCTGCTGCTAG** | GTATTGAGTTGGGCTATACGGTAATTATGTCACGGCTAAAATTTTCATTA**CCTCGAGGCCAGAAGAC** |
| Nup60^S89A^-myc9* | GGAGGTTATTTCCATTCTGAGATATCCCCAGATTCTACTGTAAACCGT**G**CCGTAGTTGTT**G**CTGCAGTGGGTG | TTATAAGAGCCGCTAAAGGT |
| SpNup60-GFP | ATGGAAAGCACCCAGGAATTACCTAAATTCTCATTTTCAGTTTTGAAGGAAGAAAAGAACCGGATCCCCGGG TTAATTAA | AATTTATCTAGTCTAAATAGATATATGCCATTGAATAAAAGTATATTAATGCCAAAAAGTGAATTCGAGCTCGTTTAAAC |
| SpNup61-GFP | AGTACTGCGGAAAAGTTATTAGCCGAATTGAATGAGAAAAAGGTCTCAAAGTCAGAGAACCGGATCCCCGGGTTAATTAA | ATGTACATTTAATAGACCAAAAATAAAGGAATAAATAGTGACTGAATCAATCACTGCTTTGAATTCGAGCTCGTTTAAAC |
| SpNup211-GFP | AAAAGACAACGTGACGATGCGAACAAAGGAGGATCCAGTTCGAACCAAAAGAAAGCAAAACGGATCCCCGGGTTAATTAA | AAATCATGTTAACTAAATATGAATAGTCCTAAGAGTGATTTATGAACCATATGAAAACATGAATTCGAGCTCGTTTAAAC |
| SpAlm1-GFP | ﻿CCTAAACGGTCCAGTTCAGACGCTGGTATGGATGTTTCCAATGATGTTAAGAAAGCCAAACGGATCCCCGGGTTAATTAA | ﻿CTGTTTACAAACTCTTAAGAAACATTAAAAAGGGCATTATACCAAAAAATTCATATTTTAGAATTCGAGCTCGTTTAAAC |
| SpNup124-GFP | TCACAAACAAATGCGCCCCCGGGCCGTAAAATTGCTGTGCCCCGAAGTCGAAGAAAACGTCGGATCCCCGGGTTAATTAA | ATCATATACCCAACCGCAATGTTTTGTCATATTGTCTTGTCAACATGTCATAATATTAATGAATTCGAGCTCGTTTAAAC |

***** NOTE: Mutagenic primer can be used to introduce two mutations simultaneously: S89A and S93A. Single mutants were distinguished from double mutants and selected based on sequencing of potential transformants. I highlighted the nucleotides deviating from WT sequence in bold font.
