# Supplementary material for: Meiotic nuclear pore complex remodeling provides key insights into nuclear basket organization
Source: J Cell Biol. 2022 Dec 14;222(2):e202204039. doi: 10.1083/jcb.202204039 (PMC9754704; doi:10.1083/jcb.202204039)
Supplement: Table S4 — shows the imaging conditions used in this study. [file JCB_202204039_TableS4.docx]

**Table S4. Imaging conditions used in this study.**

| **Figure** | **RFP** | **GFP** | **POL** | **Time Interval**  **(for live imaging)** | **Sectioning** |
| --- | --- | --- | --- | --- | --- |
| 1C (Nup60, Nup2, Nup1, Mlp1, Nup49[rep1], Nup120[rep1], Nup170), 1D, 1E, 3A, 3B, 6A, 6B, 6C, 7B, S1A, S1B, S1C, S1F, S2A, S2B, S2C, S2D, S2E, S5B, S5D, S5F, S5H | 10%T, 0.025s  EX: 575/25  EM: 632/60 | 10%T, 0.025s  EX: 475/28  EM: 523/36 | 32%T, 0.1s | 5 minutes | 1 μm, 8 sections |
| 3E, 3F, 3G, 3H, 5D, 5E, 7C, 7D, 7F, 8A, 8B, 8C, 8D, 8E, 8F, 9E, 9H, S4B, S6D, S6E, S6F, S6G, S7A, S7B, S7C, S7D, S7E, S7F, S7G, S7H, S7I | 10%T, 0.025s  EX: 575/25  EM: 632/60 | 10%T, 0.025s  EX: 475/28  EM: 523/36 | 32%T, 0.1s | 15 minutes | 1 μm, 8 sections |
| 1C (Nup49[rep2], Nup120[rep2], Pom34), 1F, S1D, S1E | 32%T, 0.025s  EX: 575/25  EM: 632/60 | 10%T, 0.025s  EX: 475/28  EM: 523/36 | 32%T, 0.1s | 5 minutes | 1 μm, 8 sections |
| 2D, 2E, 2F, 3C, 3D, 7E, S3B, S3C, S3D, S4A | 32%T, 0.025s  EX: 575/25  EM: 632/60 | 10%T, 0.025s  EX: 475/28  EM: 523/36 | 32%T, 0.1s | 15 minutes | 1 μm, 8 sections |
| S3A | 10%T, 0.025s  EX: 575/25  EM: 632/60 | 32%T, 0.025s  EX: 475/28  EM: 523/36 | 32%T, 0.1s | 15 minutes | 1 μm, 8 sections |
| S3F, S3G | 32%T, 0.025s  EX: 575/25  EM: 632/60 | 32%T, 0.025s  EX: 475/28  EM: 523/36 | 32%T, 0.1s | 15 minutes | 1 μm, 8 sections |
| 9C, 9D, 9F, 9G | 10%T, 0.025s  EX: 575/25  EM: 632/60 | 10%T, 0.025s  EX: 475/28  EM: 523/36 | 32%T, 0.1s | N/A | 1 μm, 8 sections |
| 10B, 10C, 10D, 10E, 10F, S8A, S8B, S8C, S8D, S8E, S8F, S8G, S9A, S9B, S9C, S9D, S9E, S9F | 50% T,  0.2s  EX: 561  EM: 605/70 | 35% T,  0.1–0.2s  EX: 488  EM: 525/36 | 0.1s | 5 minutes | 0.5 um, 17 sections |

All z-projections were maximum intensity projections over the entire range of acquisition
