# Supplementary material for: Meiotic nuclear pore complex remodeling provides key insights into nuclear basket organization
Source: J Cell Biol. 2022 Dec 14;222(2):e202204039. doi: 10.1083/jcb.202204039 (PMC9754704; doi:10.1083/jcb.202204039)
Supplement: Table S7 — lists sporulation efficiency for various alleles in this study. [file JCB_202204039_TableS7.docx]

**Table S7. Sporulation efficiency for various alleles in this study.** Sporulation efficiency was assessed by scoring at least 299 cells after ~24 hours in SPM. For *FKBP12-NUP60-GFP SEH1-FRB* tether strains, either DMSO (control) or 10 µM rapamycin (to induce FKBP12-FRB dimerization) was added after 4 hours in SPM. All tether strains had Pom34-mCherry and *fpr1Δ* in the background.

| **Strain** | **Unsporulated** | **Dyads** | **Triads/**  **Tetrads** |
| --- | --- | --- | --- |
| Wild type - replicate #1  UB15 | 4.3% | 0.0% | 95.7% |
| Wild type – replicate #2  UB15 | 5.3% | 0.0% | 94.7% |
| *NUP60-9A* – diploid #1  UB31853 | 4.7% | 0.7% | 94.7% |
| *NUP60-9A* – diploid #2  UB31854 | 10.0% | 0.7% | 89.3% |
| *NUP60-ΔAH* – diploid #1  UB31855 | 6.7% | 0.3% | 93.0% |
| *NUP60-ΔAH* – diploid #1  UB31856 | 8.0% | 2.3% | 89.7% |
| *FKBP12-NUP60-GFP SEH1-FRB* + DMSO – diploid #1  UB27298 | 8.0% | 1.5% | 90.5% |
| *FKBP12-NUP60-GFP SEH1-FRB* + DMSO – diploid #2  UB27299 | 7.7% | 5.4% | 87.0% |
| *FKBP12-NUP60-GFP SEH1-FRB* + rapamycin – diploid #1  UB27298 | 8.5% | 2.3% | 89.3% |
| *FKBP12-NUP60-GFP SEH1-FRB* + rapamycin – diploid #2  UB27299 | 7.3% | 5.0% | 87.7% |
| *FKBP12-NUP60-GFP* only  + DMSO – diploid #1  UB34204 | 8.8% | 3.8% | 87.5% |
| *FKBP12-NUP60-GFP* only  + DMSO – diploid #2  UB34205 | 9.3% | 4.0% | 86.7% |
| *FKBP12-NUP60-GFP* only  + rapamycin – diploid #1  UB34204 | 9.0% | 2.8% | 88.3% |
| *FKBP12-NUP60-GFP* only  + rapamycin – diploid #2  UB34205 | 10.3% | 4.7% | 85.0% |
