# Supplementary material for: Meiotic nuclear pore complex remodeling provides key insights into nuclear basket organization
Source: J Cell Biol. 2022 Dec 14;222(2):e202204039. doi: 10.1083/jcb.202204039 (PMC9754704; doi:10.1083/jcb.202204039)
Supplement: Table S8 — lists gamete viability for various alleles in this study. [file JCB_202204039_TableS8.docx]

**Table S8. Gamete viability for various alleles in this study.** Gamete viability was assessed by digesting tetrads with 0.1 mg/mL zymolyase for 10 minutes, quenching with 1 mL water, dissecting onto YPD plates, and assessing colony presence after at least 48 hours of growth. For non-tether strains, diploids were incubated on SPM plates for 48 hours prior to dissection. For *FKBP12-NUP60-GFP SEH1-FRB* tether strains, diploids were incubated for 24 hours in liquid SPM, with either DMSO (control) or 10 µM rapamycin (to induce FKBP12-FRB dimerization) added after 4 hours. Cells were washed in 1 mL MilliQ water prior to dissection to ensure that the treatments had no effect on gamete viability. All tether strains had Pom34-mCherry and *fpr1Δ* in the background.

| **Strain** | **Spores Dissected** | **Gamete Viability** |
| --- | --- | --- |
| Wild type  UB15 | 576 | 99.5% |
| *NUP60-9A* – diploid #1  UB31853 | 288 | 99.7% |
| *NUP60-9A* – diploid #2  UB31854 | 288 | 98.6% |
| *NUP60-ΔAH* – diploid #1  UB31855 | 288 | 98.3% |
| *NUP60-ΔAH* – diploid #2  UB31856 | 288 | 100% |
| *FKBP12-NUP60-GFP SEH1-FRB* + DMSO – diploid #1  UB27298 | 192 | 95.8% |
| *FKBP12-NUP60-GFP SEH1-FRB* + DMSO – diploid #2  UB27299 | 192 | 89.6% |
| *FKBP12-NUP60-GFP SEH1-FRB* + rapamycin – diploid #1  UB27298 | 192 | 96.4% |
| *FKBP12-NUP60-GFP SEH1-FRB* + rapamycin – diploid #2  UB27299 | 192 | 93.2% |
| *FKBP12-NUP60-GFP* only  + DMSO – diploid #1  UB34204 | 192 | 99.5% |
| *FKBP12-NUP60-GFP* only  + DMSO – diploid #2  UB34205 | 192 | 96.4% |
| *FKBP12-NUP60-GFP* only  + rapamycin – diploid #1  UB34204 | 192 | 95.3% |
| *FKBP12-NUP60-GFP* only  + rapamycin – diploid #2  UB34205 | 192 | 99.0% |
