# Supplementary figures and images for: Meiotic nuclear pore complex remodeling provides key insights into nuclear basket organization
Source: J Cell Biol. 2022 Dec 14;222(2):e202204039. doi: 10.1083/jcb.202204039 (PMC9754704; doi:10.1083/jcb.202204039)

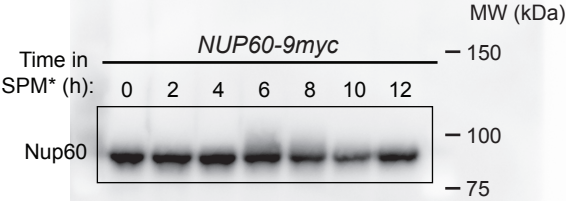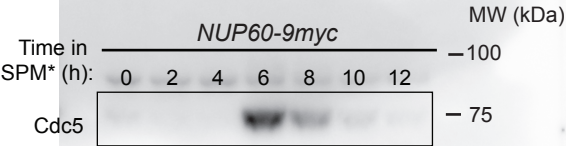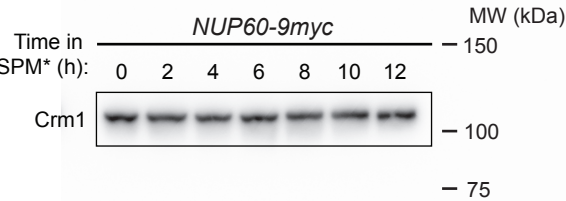

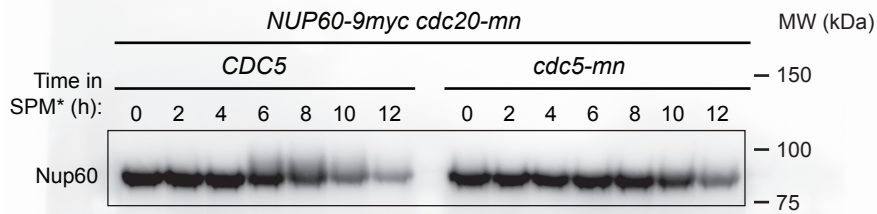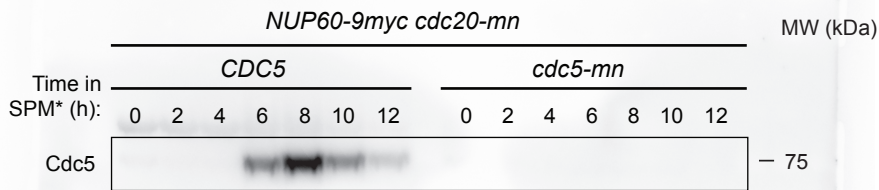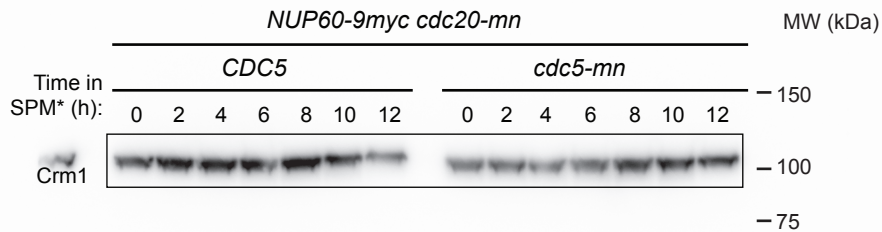

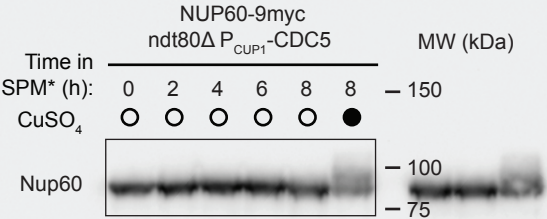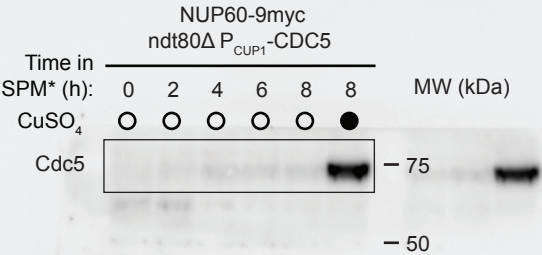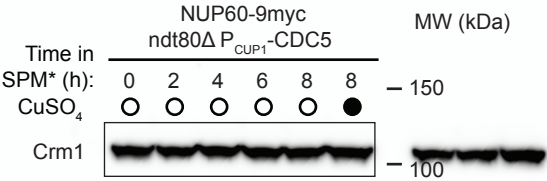

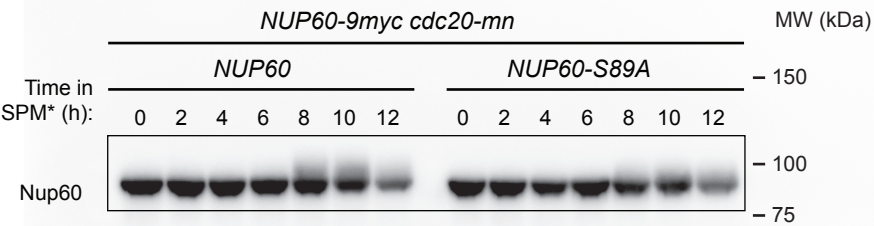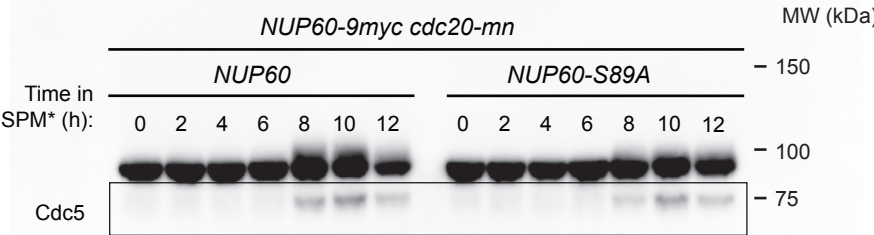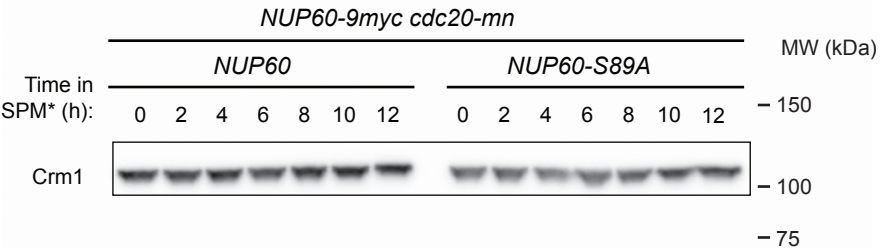

Supplement: SourceData F4 — is the source file for Fig. 4. [file JCB_202204039_SourceDataF4.pdf]

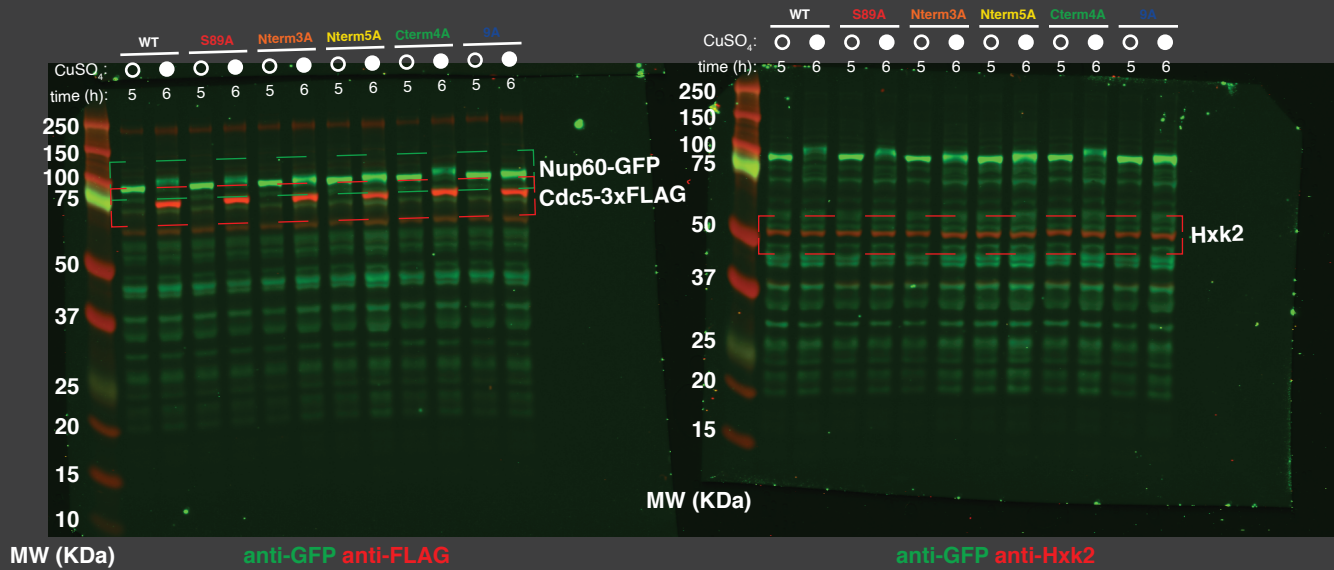

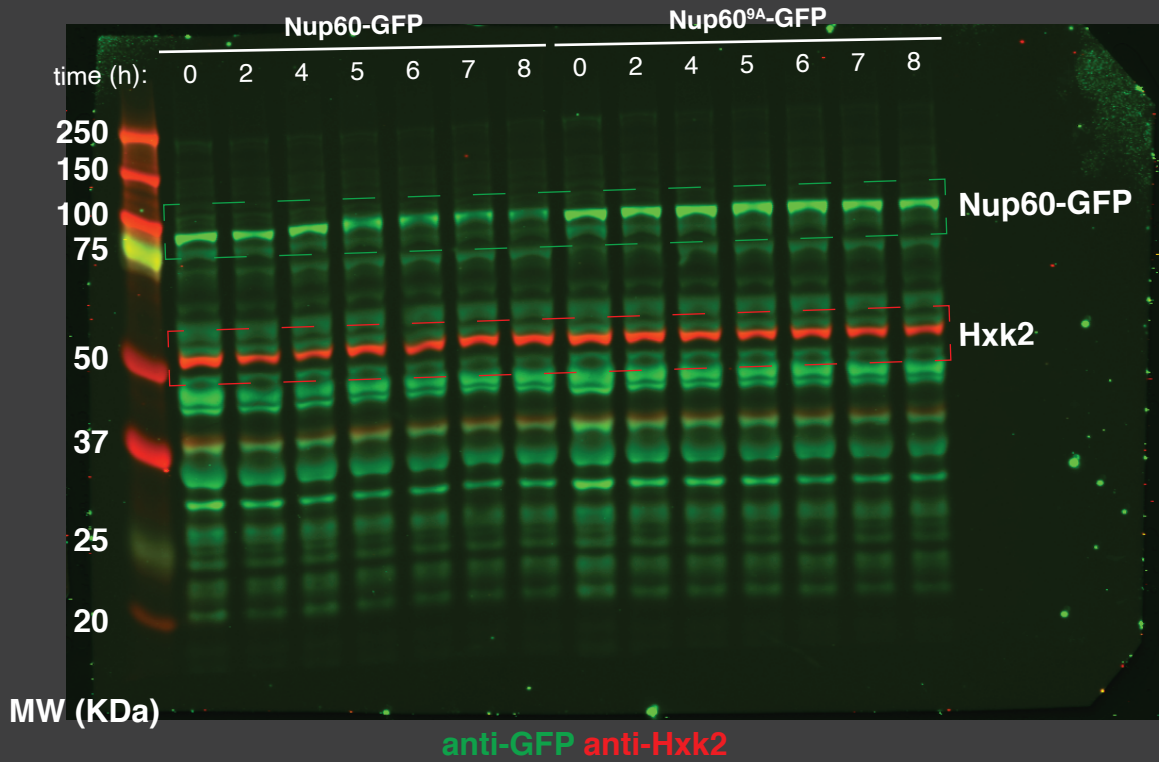

Supplement: SourceData F5 — is the source file for Fig. 5. [file JCB_202204039_SourceDataF5.pdf]

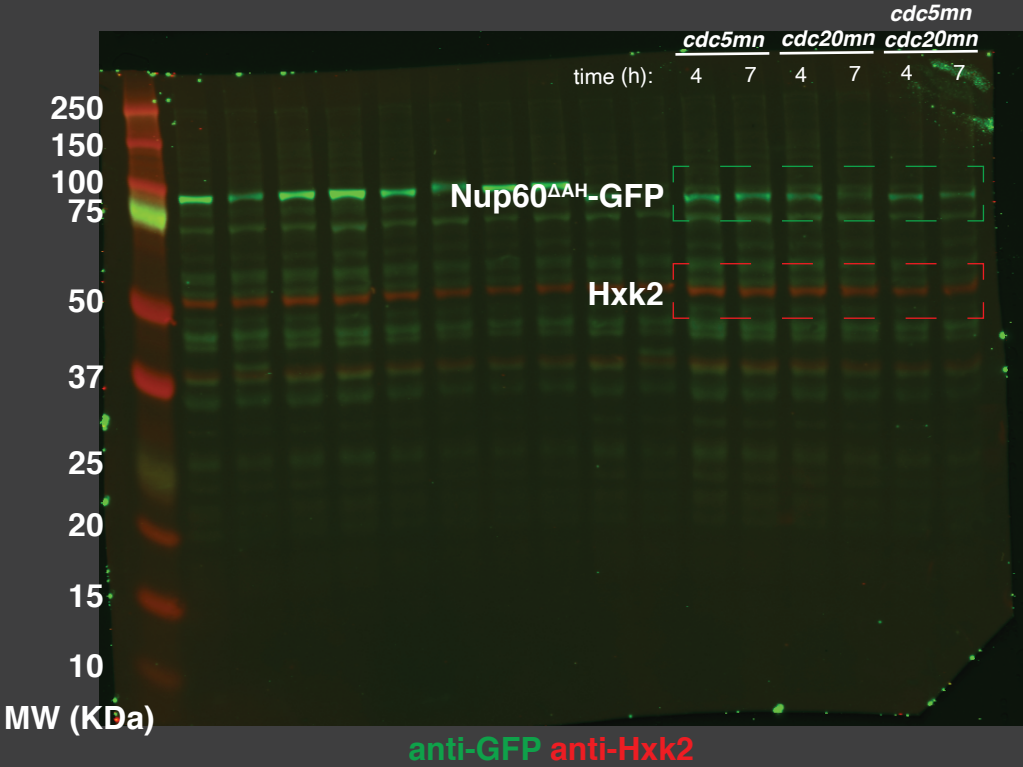

Supplement: SourceData F7 — is the source file for Fig. 7. [file JCB_202204039_SourceDataF7.pdf]

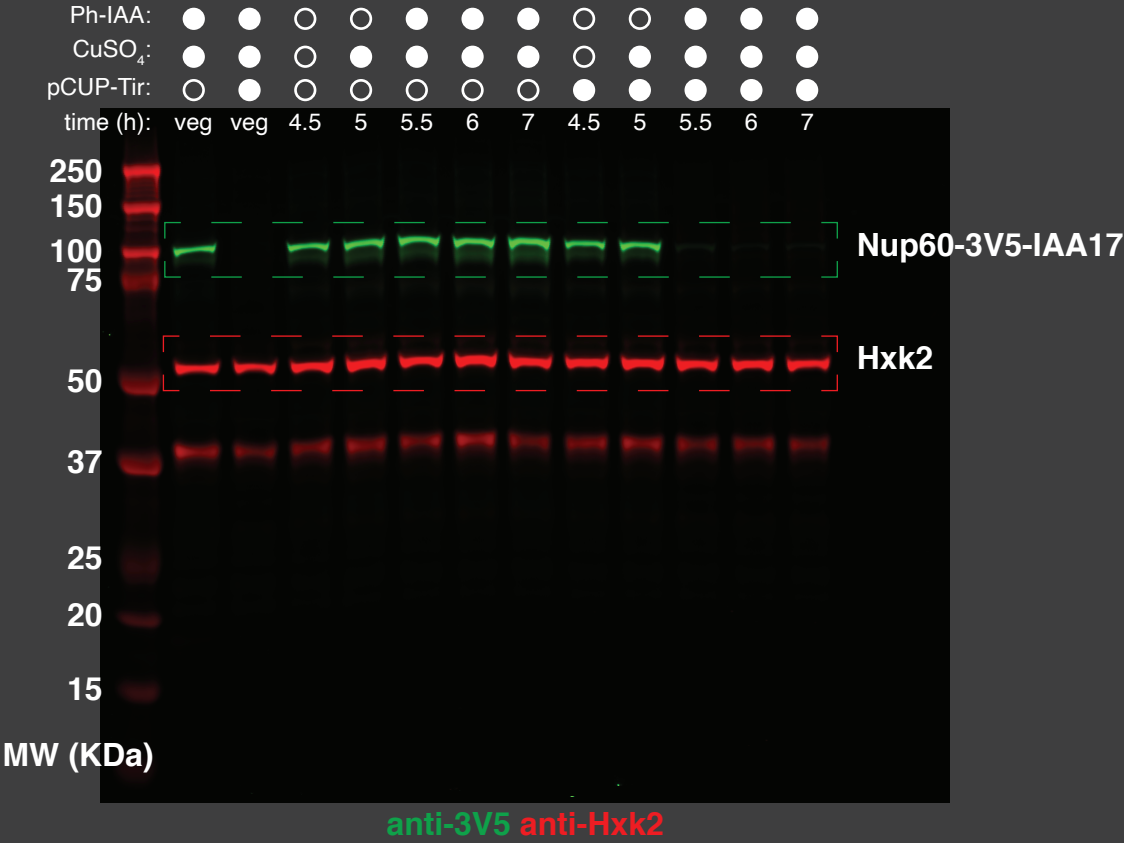

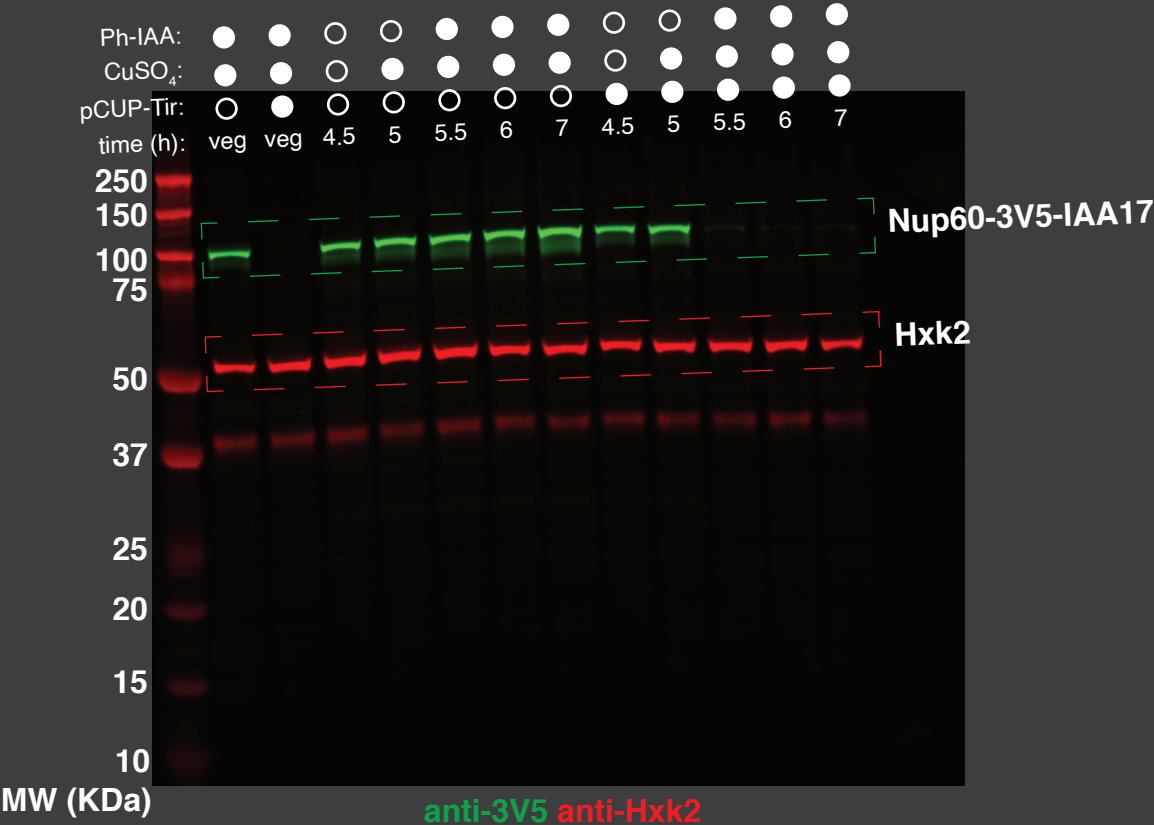

Supplement: SourceData F9 — is the source file for Fig. 9. [file JCB_202204039_SourceDataF9.pdf]

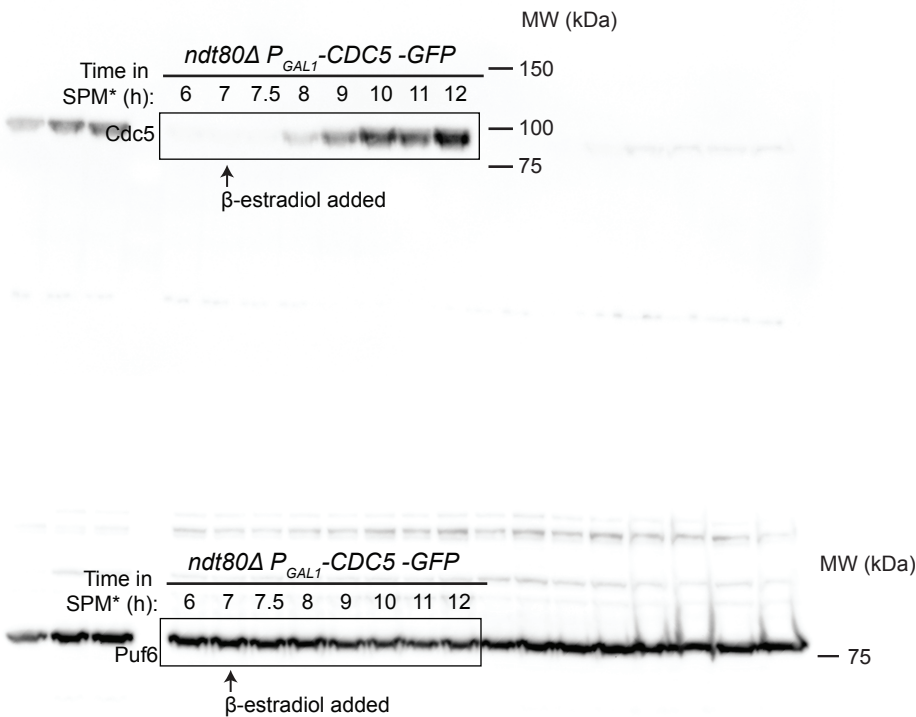

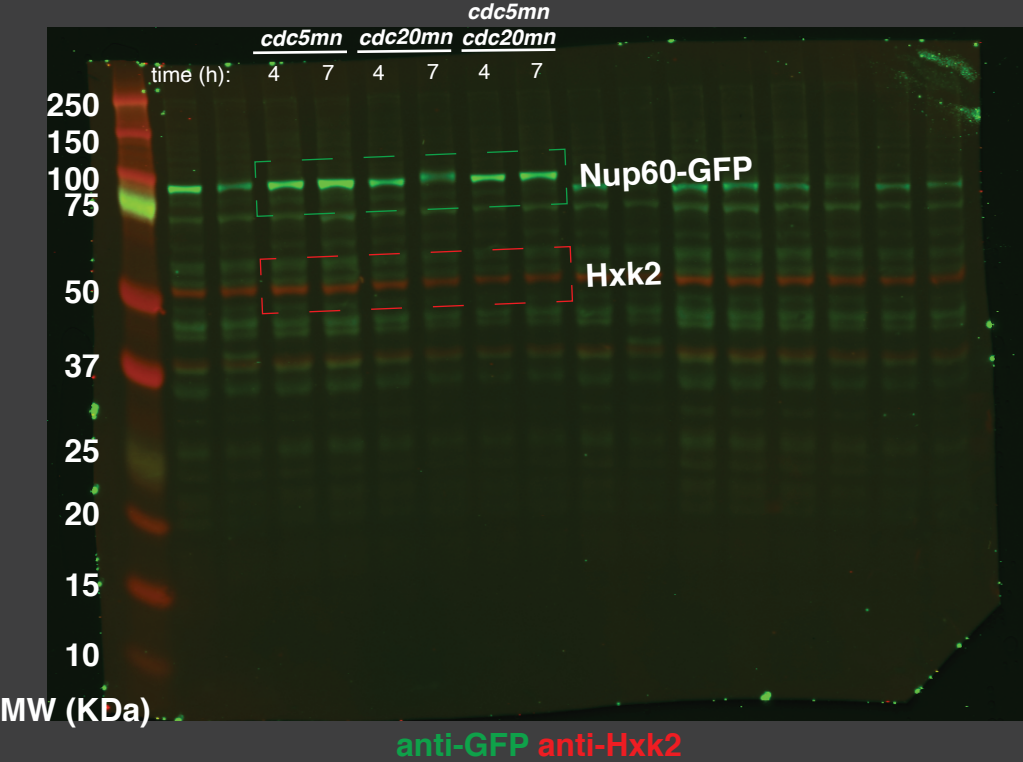

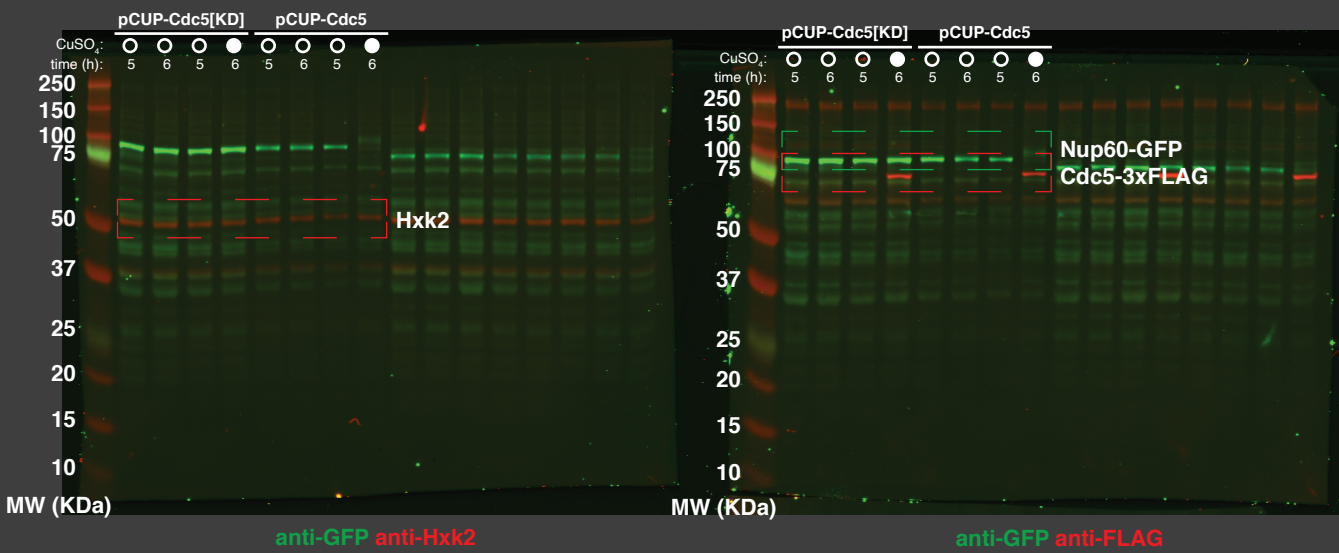

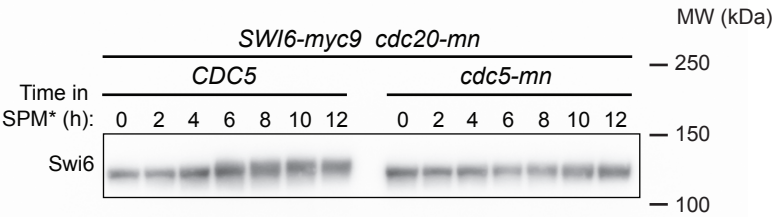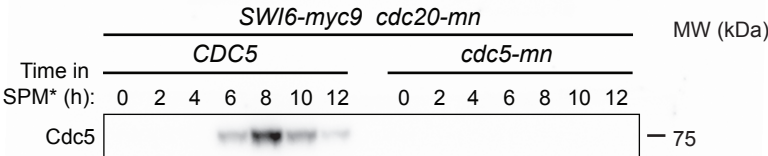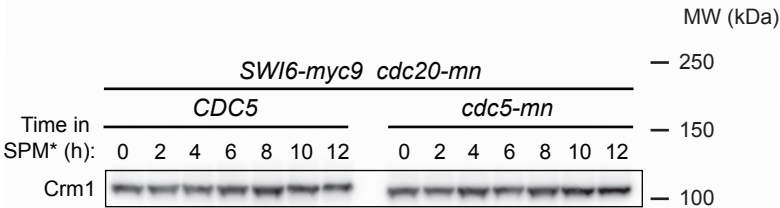

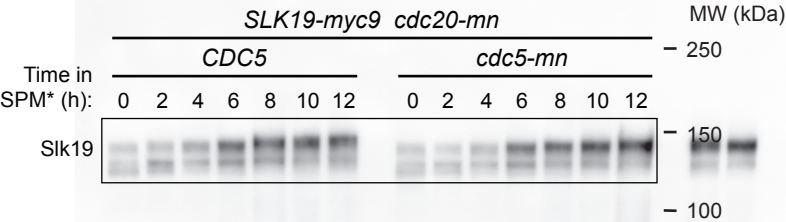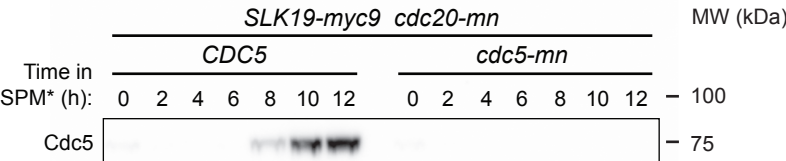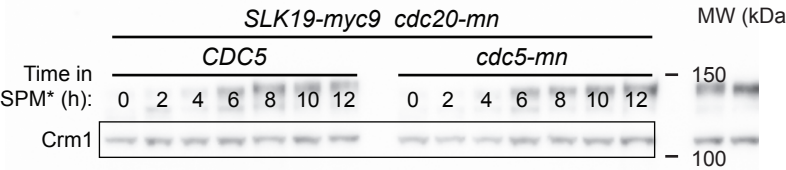

Supplement: SourceData FS4 — is the source file for Fig. S4. [file JCB_202204039_SourceDataFS4.pdf]

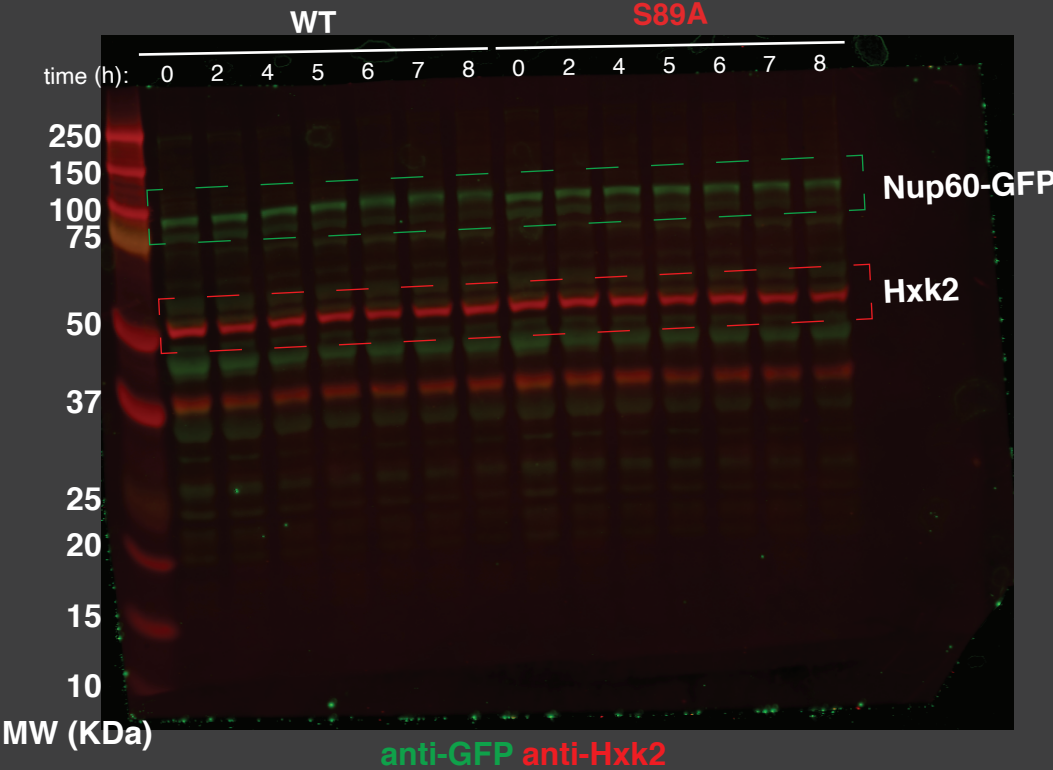

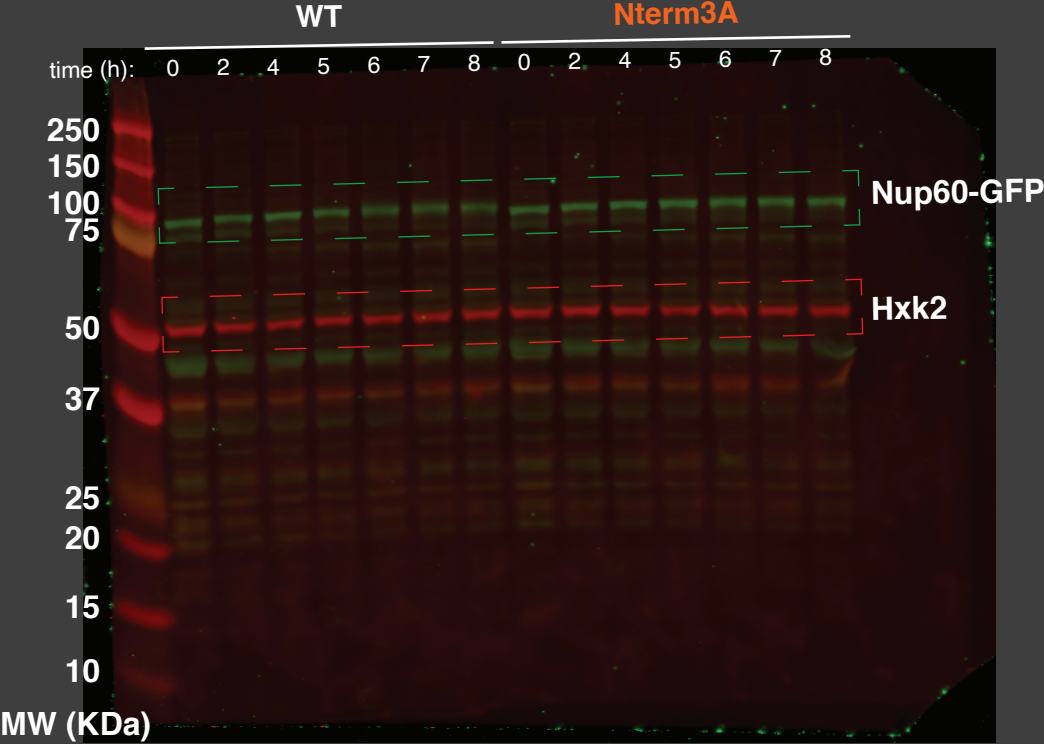

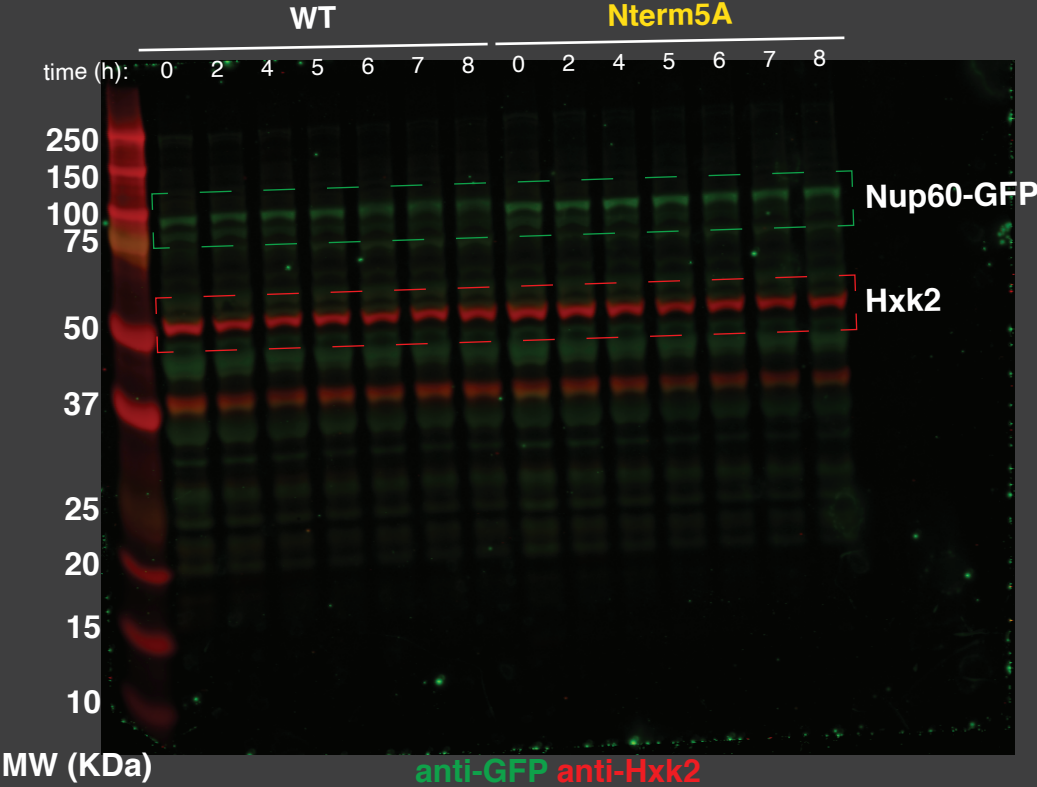

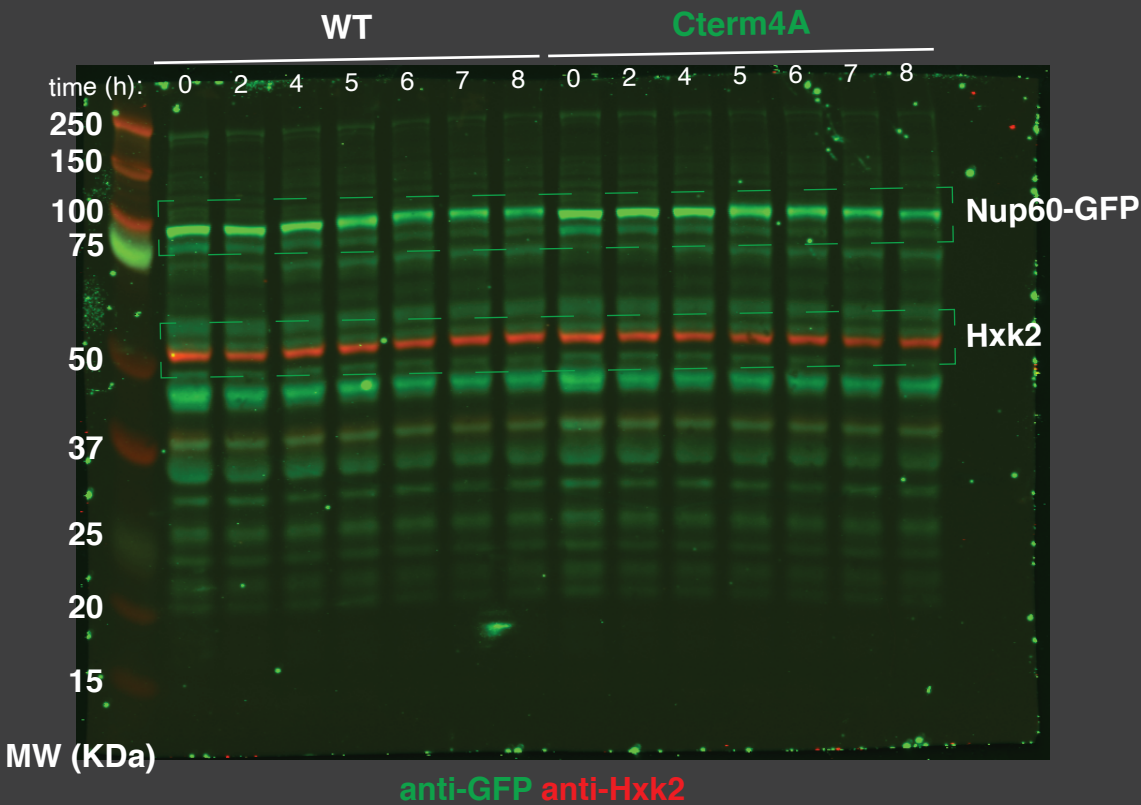

Supplement: SourceData FS5 — is the source file for Fig. S5. [file JCB_202204039_SourceDataFS5.pdf]

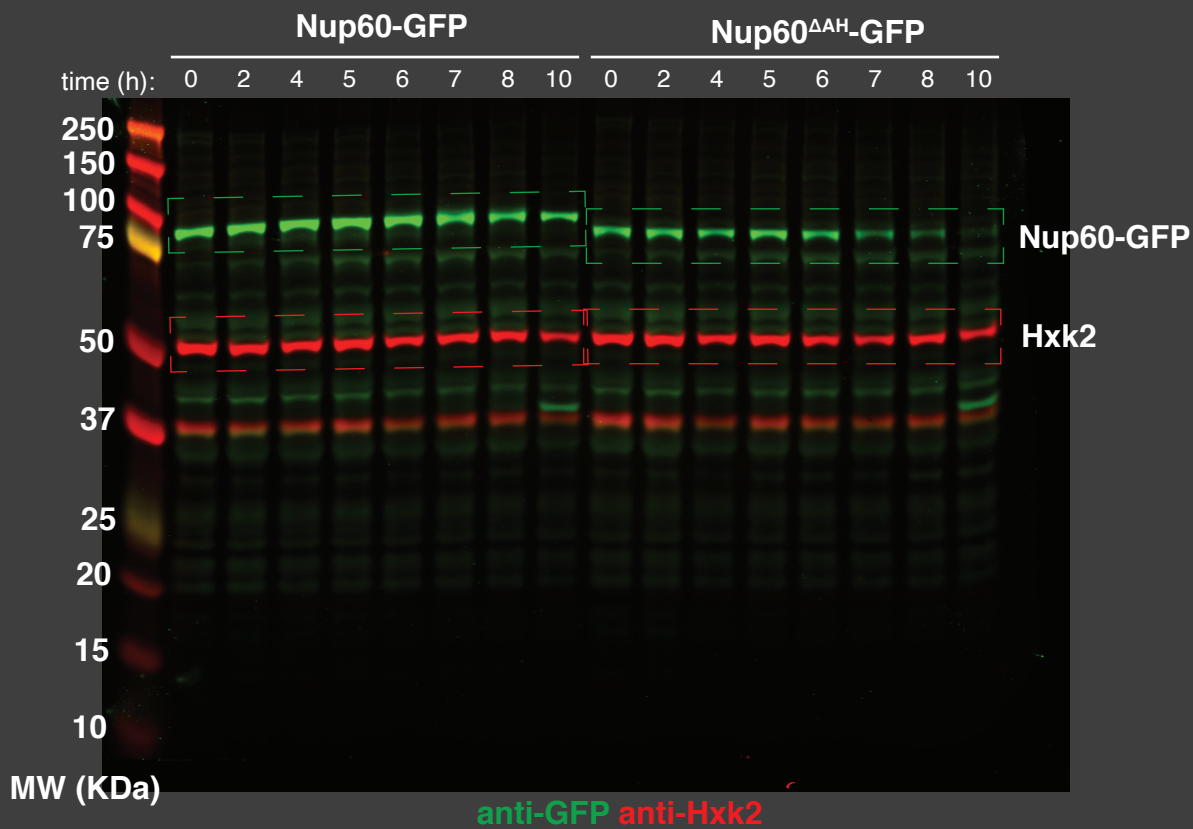

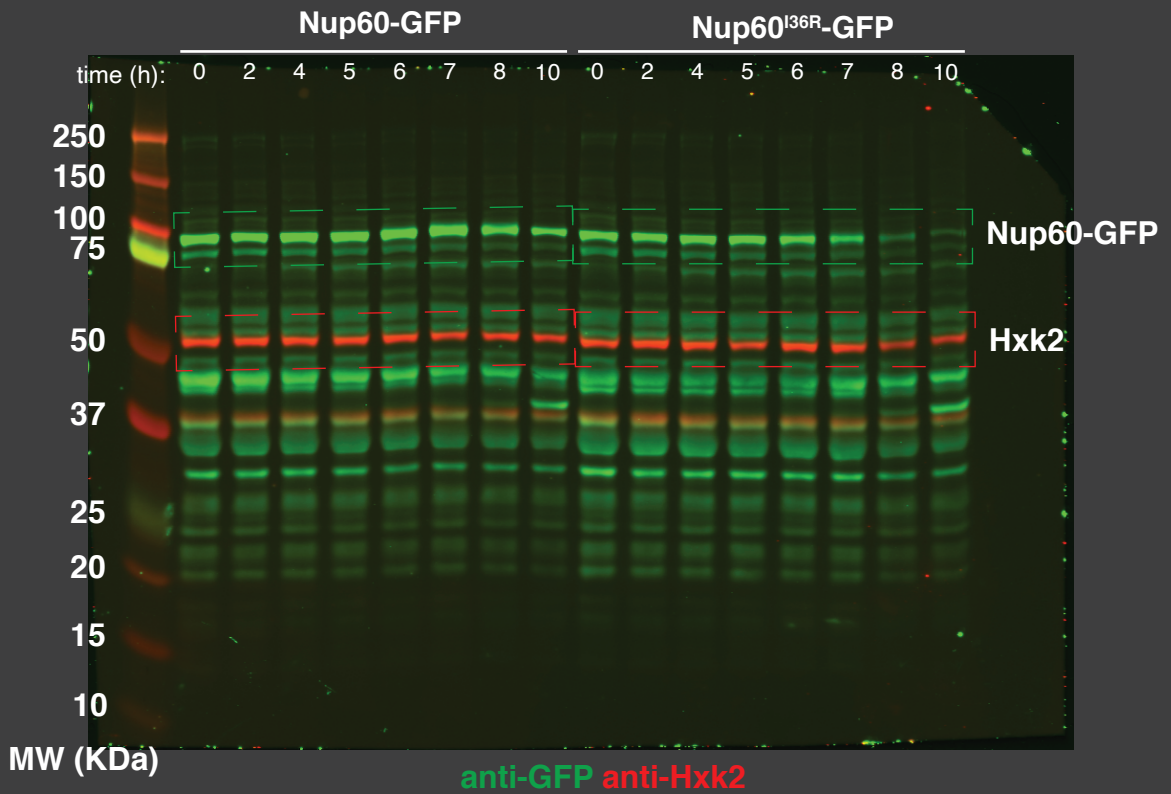

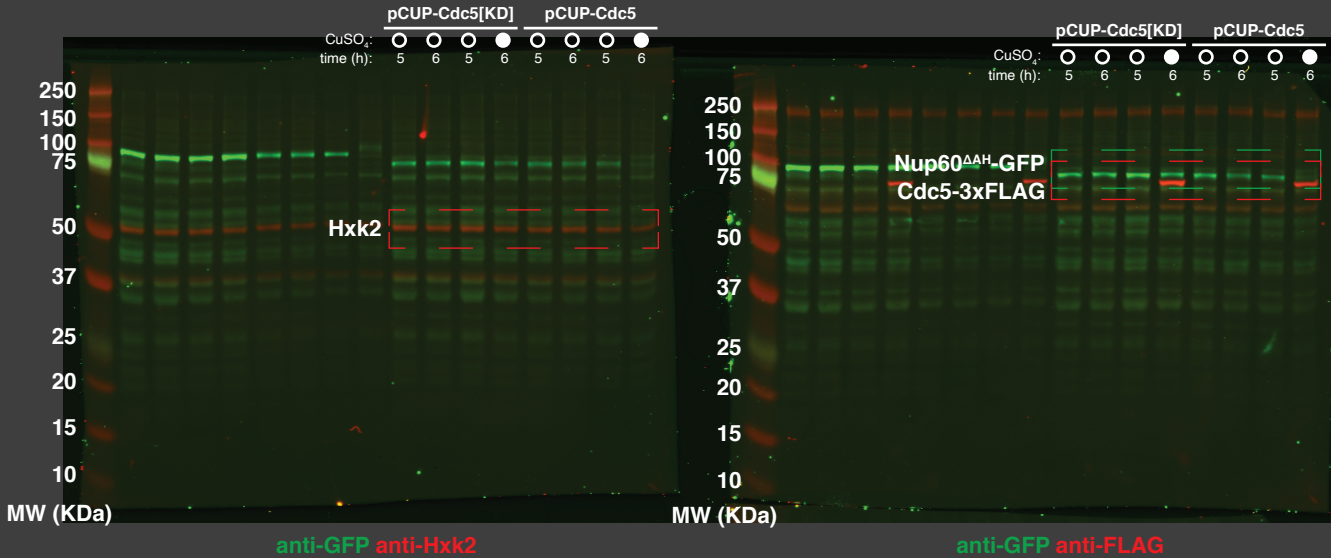

Supplement: SourceData FS6 — is the source file for Fig. S6. [file JCB_202204039_SourceDataFS6.pdf]
